# Moral Distress Among Family Caregivers: A Concept Analysis

**DOI:** 10.1111/jan.70444

**Published:** 2026-01-03

**Authors:** Tharaa Ananzeh, Caroline F. Morrison, Elaine L. Miller, Natalie Kreitzer, Tamilyn Bakas

**Affiliations:** ^1^ College of Nursing University of Cincinnati Cincinnati Ohio USA; ^2^ College of Medicine University of Cincinnati Cincinnati Ohio USA

**Keywords:** caregiving burden, decision‐making, ethical dilemmas, family caregivers, internal conflict, moral distress

## Abstract

**Aim:**

To examine and define the concept of moral distress among family caregivers by identifying its key attributes, antecedents and consequences.

**Design:**

Concept analysis.

**Methods:**

This study was guided by Walker and Avant's concept analysis framework. A comprehensive literature search was conducted to identify relevant studies, with 12 articles included in this analysis.

**Data Sources:**

PubMed, CINAHL, Scopus and PsycINFO databases were searched for articles published between February 2000 and May 2025.

**Results:**

Three defining attributes of moral distress in family caregivers were identified: self‐directed negative emotions, internal conflict and feelings of powerlessness and helplessness. Antecedents included caregiving burden, role conflict, ethical dilemmas, complex decision‐making and internal and external constraints. Consequences encompassed long‐term health effects, social withdrawal, burnout and moral residue. These findings led to a conceptual definition of moral distress in family caregivers.

**Conclusion:**

Moral distress in family caregivers is a significant and underrecognised issue that affects caregiver well‐being and the quality of care they provide. This concept analysis offers a clear conceptual definition, providing a foundation for developing research instruments and interventions.

**Implications for the Profession and/or Patient Care:**

Healthcare professionals should recognise moral distress in family caregivers as a key factor impacting both caregiver well‐being and patient care. Support through education, counselling and peer groups can reduce moral distress and foster more ethical, collaborative care environments.

**Impact:**

This study addressed the lack of clarity surrounding moral distress in family caregivers. It identified key attributes, antecedents, and consequences, and developed a clear conceptual definition. These insights will inform research, practice and policy. The findings will benefit caregivers, improve patient care and support healthcare teams.

**Reporting Method:**

This study followed Walker and Avant's framework and employed the Preferred Reporting Items for Systematic Reviews and Meta‐Analyses (PRISMA) guidelines in article selection.

**Patient or Public Contribution:**

No patient or public involvement.

## What Does This Paper Contribute to the Wider Global Clinical Community?

- Clarifies the concept of moral distress in family caregivers, a population often overlooked in moral distress research.
- Identifies specific attributes, antecedents, and consequences of moral distress in caregiving, providing insight for targeted support interventions.
- Highlights the need for healthcare professionals to recognise and address family caregivers' moral distress to improve caregivers' well‐being and patient outcomes.

## 1 Introduction

Family caregivers, also called informal caregivers, are individuals who provide regular care and assistance to friends, neighbours or family members with health conditions requiring support (Centers for Disease Control and Prevention 2018). According to the Family Caregiver Alliance, there are two types of family caregivers, primary or secondary, depending on their level of involvement in caregiving responsibilities. Both primary and secondary caregivers can live with or separately from the person they assist (Family Caregiver Alliance, n.d.). In 2020, the National Alliance for Caregiving (NAC) and AARP reported that approximately 53 million Americans (24%) served as family caregivers, which has risen significantly from 43.5 million (18%) in 2015. Additionally, 61% of these caregivers were employed while providing care. Globally, the International Alliance of Carer Organisations estimates that there are over 63 million family carers worldwide (International Alliance of Carer Organisations 2018). Family caregiving is also increasingly recognised as a significant global health concern. In 2025, the World Health Organisation signed a partnership to strengthen support for family caregivers worldwide, underscoring the essential but often overlooked role of families in health systems (World Health Organisation 2025).

Family caregivers work as an extension to the health care institutions, performing complex medical tasks and providing care based on the specific needs of the illness (American Psychological Association 2011). They support individuals with acute and chronic conditions, including Alzheimer's, stroke, heart disease, cancer, mental health disorders, developmental disabilities and mobility issues (AARP and National Alliance for Caregiving 2020). Among the many caregiving populations, those caring for patients with dementia and Alzheimer's disease often face additional challenges due to the complex symptoms of the disease (Lindeza et al. 2024). They assist with instrumental activities of daily living, personal activities of daily living, medication management and adherence and managing behavioural symptoms such as aggression and depression (Alzheimer's Association 2024). Those taking care of stroke survivors face unique challenges as well (Martin et al. 2024). The caregiving role of stroke survivors involves many tasks, including providing physical and personal support, administering medication, managing follow‐up appointments, ensuring safety and providing both emotional and financial support (American Stroke Association 2023). In contrast to stroke caregiving, caring for cancer patients presents its own unique set of challenges (Schulz and Eden 2016). One of these challenges is caring for a person with cancer at the end‐of‐life while facing low mental and physical health (National Cancer Institute 2024). This includes managing severe pain, coordinating palliative care and dealing with anticipatory grief (Bilić et al. 2022).

As a result of these responsibilities, caregivers often experience significant burdens (Jaracz et al. 2024; Sheehan et al. 2021). These include emotional distress (McCurley et al. 2019; Sugawara et al. 2023), stress (Lewandowska 2022; Sajwani‐Merchant et al. 2023; Vu et al. 2022), depression (Gurley Nettles 2024; Sheehan et al. 2021) and role conflict distress (MacKenzie 2024). Moreover, they may experience physical health problems (Sugawara et al. 2023; Vu et al. 2022), and feelings of frustration, guilt, and powerlessness are also common (McCurley et al. 2019; Shewangizaw et al. 2023). These burdens create internal conflicts, decision‐making challenges (Lawson 2022) and ethical dilemmas among family caregivers (Koenig 2004; Lafrance et al. 2022; Stork et al. 2018). Unlike healthcare professionals, family caregivers often lack sufficient education and training in caregiving, and this makes it challenging for them to deal with difficult situations (Lobo et al. 2021). All these challenges, burdens, feelings and ethical dilemmas make it difficult for caregivers to make decisions that align with their moral values and beliefs (James 2021).

## 2 Background

Moral distress occurs when family caregivers are forced to make decisions that conflict with their ethical beliefs and values due to a number of reasons, such as lack of resources, support, knowledge or time (Ullrich et al. 2020). As a result of the difficult decisions, family caregivers often experience negative emotional, physical and psychological effects, including guilt, frustration, powerlessness, stress, regret and disturbed sleep (Ullrich et al. 2020). The concept of moral distress was first introduced in nursing by Jameton (1984), who defined it as the distress that occurs when individuals recognise the ethically right course of action to take but are unable to act due to various constraints. Moral distress has been frequently examined among healthcare professionals, particularly nurses, who face institutional barriers that prevent them from providing optimal patient care (Epstein and Hamric 2009). There is agreement that moral distress is a fluid and context‐dependent experience (Hardingham 2004; Wilkinson 1987). The variability in its attributes across different contexts highlights its dynamic nature and fluidity (Epstein and Hamric 2009). As every community has its unique context and characteristics, it is important to study this concept in different communities, such as family caregivers.

Before studies can be conducted on moral distress among family caregivers, a clear definition of moral distress in the context of family caregiving is needed. Without a clear conceptual definition, researchers cannot effectively measure, study, or develop interventions to support family caregivers experiencing moral distress. The conceptual definition will facilitate the identification of the operational definition of moral distress, features and key elements among family caregivers. As a result, this contributes to the advancement of general knowledge and a more comprehensive understanding of the concept of moral distress. A comprehensive review of the literature reveals that the concept of moral distress among family caregivers has not been thoroughly examined or conceptualised. Thus, the purpose of this paper is to rigorously examine and define the concept of moral distress among family caregivers by identifying its key attributes, antecedents and consequences.

## 3 Methods (Data Sources)

### 3.1 Design

This concept analysis follows Walker and Avant's (2019) method, which provides a structured and systematic approach to clarifying and defining moral distress in the context of family caregiving. The results of the concept analysis provide comprehensive, clear, and precise definitions and give a basic understanding of the concept's characteristics and underlying attributes. Additionally, the findings can help researchers develop new research instruments, improve existing tools and refine interview questions (Walker and Avant 2019).

**FIGURE 1:**
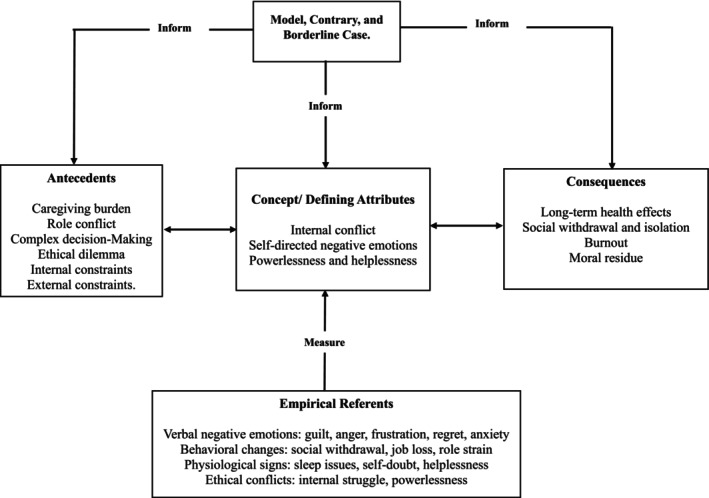
Conceptual model of moral distress in family caregivers, adapted from Walker and Avant (2019).

Walker and Avant's concept analysis method is an updated version of Wilson's (1963) classic concept analysis. Walker and Avant's method has eight iterative steps: (1) selecting a concept, (2) determining the aim of the analysis, (3) identifying the uses of the concept, and (4) defining its key attributes. The process continues with (5) developing a model case, (6) presenting a borderline and contrary case, (7) identifying antecedents and consequences and (8) concluding with empirical referents. Figure 1 represents the concept analysis process, adapted from Walker and Avant (2019).

### 3.2 Literature Search

A comprehensive literature search was conducted across four databases: PubMed, CINAHL, Scopus and PsycINFO. The search used both MeSH terms and keywords to identify articles relevant to this concept analysis. Keywords include family caregiver, informal caregiver, morality, non‐professional caregiver, moral distress, ethical dilemma, caregiver guilt, powerlessness, ethical conflict, decision‐making and home care. The Boolean operators ‘AND’ and ‘OR’ were used to refine the search strategy. As most research on moral distress has focused on healthcare professionals, we employed a comprehensive and multi‐database strategy to capture the full scope of evidence and to strengthen methodological rigour.

The search was limited to articles published between February 2000 and May 2025 to ensure a comprehensive review of research on moral distress in family caregivers. The complete search strings for each database are presented in Table 1. The inclusion criteria for this concept analysis focused on studies that (1) examined family caregivers, informal caregivers, or non‐professional caregivers, (2) addressed moral distress within the context of family caregiving for individuals with acute and chronic illnesses (e.g., stroke, brain injury, dementia, cancer and Alzheimer's), (3) included discussions of ethical dilemmas, guilt, powerlessness, or related concepts; and (4) are published in peer‐reviewed journals. Articles that focused on health care professionals (e.g., nurses, physicians) or were not published in English were excluded.

**TABLE 1:** Search strategy.

Database	Search string
PubMed	((‘Caregivers’[MeSH] OR ‘family caregiver*’[tiab] OR ‘informal caregiver*’[tiab] OR ‘non‐professional caregiver*’[tiab]) AND (‘moral distress’[tiab] OR ‘ethical dilemma*’[tiab] OR ‘caregiver guilt’[tiab] OR ‘powerlessness’[tiab] OR ‘ethical conflict*’[tiab] OR ‘ethical stress*’[tiab] OR ‘decision making’[tiab])) AND (‘caregiving burden’[tiab] OR ‘family support’[tiab] OR ‘home care’[tiab]) NOT (‘nurses’[tiab] OR ‘physicians’[tiab] OR ‘healthcare professionals’[tiab])
Scopus	(TITLE‐ABS‐KEY (‘family caregiver*’ OR ‘informal caregiver*’ OR ‘non‐professional caregiver*’) AND TITLE‐ABS‐KEY (‘moral distress’ OR ‘ethical dilemma*’ OR ‘caregiver guilt’ OR ‘powerlessness’ OR ‘ethical conflict*’ OR ‘ethical stress*’ OR ‘decision making’) AND TITLE‐ABS‐KEY (‘caregiving burden’ OR ‘family support’ OR ‘home care’) AND NOT TITLE‐ABS‐KEY (‘nurses’ OR ‘physicians’ OR ‘healthcare professionals’))
CINAHL	(MH ‘Caregivers’ OR TX ‘family caregiver*’ OR TX ‘informal caregiver*’ OR TX ‘non‐professional caregiver*’) AND (TX ‘moral distress’ OR TX ‘ethical dilemma*’ OR TX ‘caregiver guilt’ OR TX ‘powerlessness’ OR TX ‘ethical conflict*’ OR TX ‘ethical stress*’ OR TX ‘decision making’) AND (TX ‘caregiving burden’ OR TX ‘family support’ OR TX ‘home care’) AND NOT (TX ‘nurses’ OR TX ‘physicians’ OR TX ‘healthcare professionals’)
PsycINFO	(DE ‘Caregivers’ OR TI ‘family caregiver*’ OR AB ‘family caregiver*’ OR TI ‘informal caregiver*’ OR AB ‘informal caregiver*’ OR TI ‘caregiver*’ OR AB ‘caregiver*’ OR TI ‘family caregiving’ OR AB ‘family caregiving’) AND (AB ‘moral distress’ OR AB ‘ethical dilemma*’ OR AB ‘caregiver guilt’ OR AB ‘powerlessness’ OR AB ‘ethical conflict*’ OR AB ‘ethical stress*’ OR AB ‘decision making’ OR AB ‘moral stress’ OR AB ‘ethical concerns’) AND (AB ‘caregiving’ OR AB ‘family care’ OR AB ‘informal care’ OR AB ‘family caregiving’ OR AB ‘caregiving experience’) NOT (TI ‘nurses’ OR AB ‘nurses’ OR TI ‘physicians’ OR AB ‘physicians’ OR TI ‘healthcare professionals’ OR AB ‘healthcare professionals’)

**FIGURE 2:**
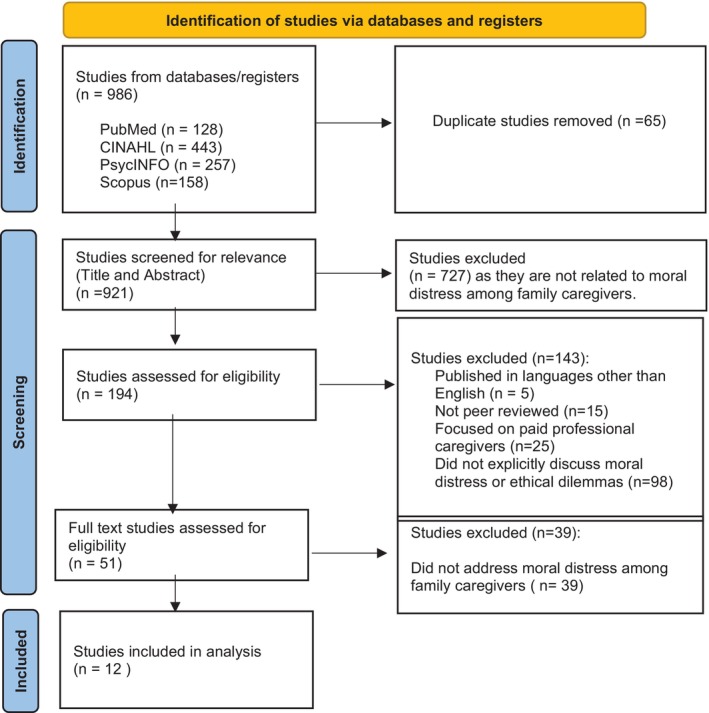
PRISMA flow chart illustrating the study selection process, adapted from Moher et al. (2009) and Page et al. (2021).

The initial search returned a total of 986 articles. Of these, 128 articles were in PubMed, 257 were in PsycINFO, 158 were in Scopus and 443 were in CINAHL. After removing duplicates and applying the eligibility criteria, 12 articles met the inclusion criteria and were included in this concept analysis. The article selection process was presented following the Preferred Reporting Items for Systematic Reviews and Meta‐Analyses (PRISMA) guidelines (Moher et al. 2009; Page et al. 2021) to ensure transparency and rigour, as illustrated in Figure 2.

We systematically reviewed these studies using Walker and Avant's framework. Specifically, the content of these articles informed the identification of defining attributes, antecedents, consequences and empirical referents of moral distress in family caregiving. The results of these steps then guided the development of the model, contrary cases and borderline cases. Collectively, these steps informed the final definition of moral distress among family caregivers. A summary of the included articles is presented in Table 2.

**TABLE 2:** Summary of the included articles.

Author	Purpose	Country	Design and sample	Definition of moral distress used	Major themes (results)	Link major themes to appropriate attributes of moral distress
Gágyor et al. (2019)	Explore ethical challenges in primary care from the perspectives of informal caregivers, general practitioners and nurses	Germany	Design: Qualitative study. Sample: Informal caregivers: 12 (spouses/children of patients). General practitioners: 11. Nurses: 14	‘Moral distress is the experience to be prevented from doing what is seen to be the right thing’ (Choe et al. 2015)	*Themes related to ethical challenges are*: Theme A: Role conflict (balancing personal, professional, and caregiving responsibilities, leading to conflicting demands and distress) Theme B: Role strain in caregiving (feeling trapped in caregiving role with high demands and lack of relief)	Theme A: Self‐directed negative emotions (frustration, guilt, anxiety) Theme B: Powerlessness/helplessness (lack of options to reduce the burden) Self‐directed negative emotions (feeling overwhelmed, frustration, guilt)
Hosseini et al. (2024)	To explore Iranian family caregivers' lived experiences of ethical decision‐making for terminally ill cancer patients	Iran	Design: Qualitative study. Sample: 12 family caregivers of end‐stage cancer patients	The study discusses ethical dilemmas but does not explicitly define moral distress	Theme A: Fluctuating between hope and despair (emotional distress, lack of preparation and support, caregiver burden) Theme B: Wandering dilemma (patient uniqueness, desire to do the right thing, struggle with futile care decisions) Theme C: Ethical decision‐making (maintaining dignity, patient best interests, adherence to tradition and religion)	Theme A: Self‐directed negative emotions (guilt, anxiety, grief) Powerlessness/helplessness Theme B: Internal conflict (tension between continuing futile treatments and respecting patient wishes) Self‐directed negative emotions Theme C: Powerlessness (caregivers felt unable to honour patient preferences due to institutional constraints and lack of resources)
Koenig (2004)	To explore the ethical dilemmas and decision‐making processes faced by women as informal caregivers of frail elders	United States	Design: Qualitative study Sample: 13 female caregivers (ages 49–85), spouses, daughters	The study does not explicitly define moral distress, but it discusses ethical dilemmas	Theme A: Types of ethical dilemmas (balancing the elder's autonomy with ensuring safety and well‐being) Theme B: Emotional responses throughout decision‐making (guilt, anxiety, frustration before, during and after decisions) Theme C: Access to informal supports and formal services (lack of supervision, respite care and financial assistance increases caregiver burdens)	Theme A: Internal conflict Self‐directed emotions. Theme B: Self‐directed emotions Internal conflict Theme C: Powerlessness/helplessness Self‐directed emotions
Kristanti and Effendy (2021)	To explore ethical dilemmas faced by family caregivers of palliative patients in Indonesia	Indonesia	Design: perspective paper based on empirical data and case studies Sample: Three case studies based on previous research (Kristanti et al. 2018, 2019) on family caregivers	The study does not explicitly define moral distress but discusses ethical dilemmas in family caregiving. It acknowledges that terms like ethics, dilemmas, and moral distress are often used interchangeably in medical literature (Yildiz 2019)	Theme A: Hiding emotions and information (caregivers suppress their feelings and sometimes withhold the diagnosis from the patient) Theme B: Voluntary vs. obligatory caregiving (caregiving seen as moral duty, causing conflicting feelings) Theme C: Lack of training and decision‐making struggles (caregivers face ethical decisions without proper guidance) Theme D: Ethical dilemmas in decision‐making (such as choices around resuscitation and treatments) Theme E: Concerns about formal facilities (cultural and societal pressure when deciding on hospice or nursing home care)	Theme A: Internal conflict (suppressing emotions to maintain a ‘cheerful’ facade) Self‐directed negative emotions (guilt, anxiety from withholding information) Theme B: Internal conflict (struggle between personal desires and familial obligations) Theme C: Powerlessness/helplessness (feeling unqualified to make critical choices) Self‐directed negative emotions (fear of harming loved ones) Theme D: Internal conflict Self‐directed negative emotions
Lafrance et al. (2022)	To explore the ethical dilemmas family caregivers face when negotiating driving safety with older adults who may no longer be fit to drive	Canada	Design: Qualitative study Sample: *N* = 15 informal caregivers of older adults with conditions impairing driving safety (dementia, Parkinson's, stroke) Caregivers of spouses, parents, or in‐laws aged 41–78 years	There is no explicit definition of moral distress, but the article focuses on an ethical dilemma that family caregivers face when intervening in driving cessation	Theme A: Gauging risk, uncertainty, and delay in intervention (caregivers struggle with the decision to intervene, balancing safety and independence) Theme B: Resistance and family conflict (caregivers experience pushback from family members or care recipients when safety is prioritised) Theme C: Ethical dilemmas and blame, between a rock and a hard place (caregivers feel morally responsible for the outcome, whether they intervene or not)	Theme A: Internal conflict Self‐directed negative emotions (fear of accidents, anxiety, frustration, guilt) Theme B: Self‐directed negative emotions (frustration, self‐blame, guilt from resistance) Theme C: Powerlessness/helplessness (lack of institutional or family support) Internal conflict (balancing safety with autonomy) Self‐directed negative emotions (fear of blame, guilt over decisions)
MacKenzie (2024)	To explore the concept of role conflict distress as a form of moral distress experienced by family caregivers when caregiving duties conflict with the norms of their loving relationships	United States	Design: Theoretical/philosophical analysis with personal narrative	Moral distress is described as the emotional discomfort that arises when individuals recognise the morally right course of action but are unable to pursue it due to internal or external constraints (Campbell et al. 2016)	Theme A: Role conflict distress (tension between caregiving and personal/loving relationship roles) Theme B: Normative conflict between roles (the expectations of being a good caregiver contradict those of being a good family member) Theme C: Reconceptualization and boundary‐setting (strategies to mitigate role conflict distress)	Theme A: Internal conflict (balancing caregiving and family roles) Self‐directed negative emotions (guilt, emotional strain) Theme B: Internal conflict (moral tension between competing roles) Self‐directed negative emotions (frustration, guilt) Powerlessness/helplessness (feeling unable to meet both role expectations) Theme C: Internal conflict (navigating emotional and relational boundaries) Powerlessness/helplessness (recognising limits in caregiving capacity)
Paidipati et al. (2023)	Explore caregivers' perceptions of benefits, burdens and moral distress of participating in cancer clinical trials	United States	Design: Qualitative descriptive study Sample: *N* = 20 caregivers, 80% spouses, 10% siblings, 5% adult children and 5% adult child‐in‐law	The article does not explicitly define moral distress	Theme A: Benefits of participating in research (improved quality of life, potential life‐saving opportunities) Theme B: Burdens of participating in research (physical, emotional and financial burdens alongside their loved one's suffering) Theme C: Family caregivers' moral distress (lack of control, decision‐making conflicts and suboptimal care)	Theme C: Powerlessness/helplessness (lack of control, frustration over delayed or inadequate care) Internal conflict (decision‐making challenges, self‐doubt) Self‐directed negative emotions (anxiety, frustration, guilt, regret, self‐doubt)
Rozo et al. (2025)	To understand events triggering moral distress among family caregivers of cancer patients in palliative care	Brazil	Design: Longitudinal qualitative study Sample: 10 family caregivers (2 male, 8 female)	Moral distress was defined in the study as feelings arising when individuals are unable to act according to their core moral values and responsibilities	Theme A: Repercussions following diagnosis (uncertainties and emotional impact immediately after cancer diagnosis) Theme B: Transformation of daily life (life changes, prioritisation, sacrifices due to caregiving responsibilities)	Theme A: Internal conflict (coping with emotional and moral uncertainty) Self‐directed negative emotions (fear, anxiety, distress) Theme B: Internal conflict (redefining roles and life priorities) Self‐directed negative emotions (guilt, frustration) Powerlessness/helplessness (loss of control over one's life and caregiving situation)
Shewangizaw et al. (2023)	Explore social and psychological consequences of stroke among family caregivers and stroke survivors in low‐income countries	Ethiopia	Design: Qualitative study Sample: *N* = 13 family caregivers and 13 stroke survivors (total 26 participants)	This article does not explicitly define moral distress. However, it describes psychological distress, frustration and emotional struggles among family caregivers	Theme A: Living on the Breadline (Financial crisis due to job loss, caregiving costs, inability to work; caregivers and stroke survivors face significant financial challenges) Theme B: Psychological and emotional toll (severe stress, fear, anxiety, suicidal thoughts due to overwhelming burden) Theme C: Elephant in the Room (social isolation and family conflict, withdrawal from social activities, strained family relationships)	Theme A: Powerlessness/helplessness (financial limitations affecting treatment and care quality) Theme B: Hopelessness Self‐directed negative emotions (fear, anger, frustration, sadness, worry) Theme C: Internal conflict (family disagreements about caregiving decisions); self‐directed negative emotions (loneliness, frustration, grief)
Ullrich et al. (2020)	To explore ethical challenges and moral distress among family caregivers of advanced cancer patients	Germany	Design: Qualitative study Sample: 12 family caregivers of advanced cancer patients (8 female, 4 males; spouses, children, friends)	Moral distress is defined as the burden or distress that people experience when they face an ethical dilemma and cannot act according to their values due to internal or external constraints (Dudzinski 2016; Jameton 1984)	Two Themes as pathways to moral distress were identified: Theme A: Ethical challenges due to complex decision making Care decisions, external stressors (e.g., lack of time, family disagreements), and situations where caregivers had to act against their own moral beliefs and values Theme B: Ethical dilemmas due to lack of decision‐making options (feeling excluded or powerless in care decisions)	Theme A: Internal conflict (complex decision‐making) Self‐directed negative emotions (anxiety, guilt, fear, regret) Theme B: Powerlessness/helplessness Self‐directed negative emotions (stress and frustration from exclusion) Internal conflict (struggle between acceptance and questioning decisions)
Vachon et al. (2023)	To explore the ethical dilemma among family caregivers during the COVID‐19 pandemic	Canada	Design: Qualitative study Sample: *N* = 20 family caregivers, 19 women, one man—Relationships: parents (15), spouses (3), siblings (1), grandparents (1)	Moral distress occurs ‘when a person faces two equally important obligations and does not know what to do, as these obligations support mutually inconsistent courses of action’ (Deschenes et al. 2020; Jameton 1984)	Theme A: Flight or fight, struggling with collective responsibility (caregivers feel torn between following public health restrictions and caregiving duties). Theme B: Being torn apart, assuming relational responsibility (balancing caregiving with obligations to other family members) Theme C: Choosing oneself, the cost of personal responsibility (ethical tension between self‐care and caregiving)	Theme A: Internal conflict (conflict between caregiving duties and public health restrictions); Powerlessness/helplessness (inability to act as desired) Theme B: Self‐directed negative emotions (feelings of guilt and emotional burden) Theme C: Internal conflict Powerlessness/helplessness Self‐directed negative emotions (guilt, regret)
Weigel (2019)	To highlight an under‐recognised domain among family caregivers of Alzheimer's patients, which is moral distress	United States	Design: Theoretical analysis using case studies Sample: Not applicable (conceptual analysis; no participants, hypothetical scenarios derived from real‐life caregiving experiences)	Draws on Jameton's (1984) definition (moral distress arises when one knows the right action but can't act due to constraints) Negative self‐directed emotions from perceived involvement in morally undesirable situations (Campbell et al. 2016)	Theme A: Guiding value‐based decisions (helping Alzheimer patients with cognitive decline to scaffold decision‐making by supporting their values) Theme B: Moral uncertainty in decision‐making (caregivers feel unsure about the right choices) Theme C: Relational and role strain (caregiving reshapes family relationships) Theme D: Under‐recognised distress (moral distress is not just for health care providers and clinicians; Family caregivers also face many ethical dilemmas)	Theme A: Internal conflict (balancing autonomy vs. influence in decisions) Powerlessness (due to medical/legal constraints) Theme B: Internal conflict (lack of confidence in decisions) Self‐directed negative emotions (self‐doubt, guilt, anxiety) Theme C: Self‐directed negative emotions (anger, guilt, frustration) Theme D: Powerlessness Self‐directed negative emotions (unacknowledged burden leading to powerlessness, frustration, anxiety)

*Note:* Themes (A, B, C, etc.) represent key findings identified in each study. Labels are for clarity only and do not indicate order or importance.

## 4 Overview of the Concept

### 4.1 The Uses of the Concept

The concept of moral distress was initially explored among healthcare professionals, especially nurses (Epstein and Hamric 2009). It was initially defined by Jameton (1984) as knowing the right course of action but not being able to do it due to institutional constraints. Building on this, Wilkinson (1987) described it as the psychological disequilibrium and negative feeling the person experiences when they make a moral decision that does not align with their values and beliefs. Corley et al. (2001) expanded this definition to include the painful feeling that results from recognising ethically correct behaviours but not doing them due to a lack of time, legal considerations and institutions' policies. More recently, Barlem and Ramos (2015) defined moral distress as a sense of powerlessness that prevents individuals from taking morally appropriate actions leading to physical, psychological and behavioural consequences.

Beyond nursing, moral distress has been studied among physicians (Miljeteig et al. 2023) and students, including both medical and nursing students (Ong et al. 2022). Additionally, the concept of moral distress has been explored among social workers, with institutional rules identified as the primary reason for their moral distress (Jaskela et al. 2018). Furthermore, research has applied and studied this concept in other contexts and populations, including schoolteachers (Thumvichit 2023). School teachers face a lot of ethical dilemmas and complex decision‐making situations, making them more susceptible to experiencing moral distress, which negatively affects their well‐being and job satisfaction (Váchová 2019).

Family caregivers are one of these populations that face numerous ethical and decision‐making challenges (Kristanti and Effendy 2021; Rozo et al. 2025; Vachon et al. 2023). Moral distress has been studied among family caregiver populations, including those who care for cancer patients (Paidipati et al. 2023; Rozo et al. 2025), and individuals with dementia and Alzheimer's disease (James 2021).

### 4.2 Determining the Defining Attributes

Defining attributes are the essential cluster of characteristics of a concept that consistently and frequently appear in the literature (Walker and Avant 2019). These attributes help distinguish a concept from other similar or related concepts. For this concept analysis of moral distress among family caregivers, three main defining attributes or characteristics were identified based on a comprehensive review of the literature: internal conflict, self‐directed negative emotions and feelings of powerlessness and helplessness.

### 4.3 Attributes

#### 4.3.1 Internal Conflict

Internal conflict occurs among family caregivers when they experience tension between their personal values, beliefs and caregiving responsibilities. Many family caregivers feel they need to hide their true emotions and show that they are positive, supportive and happy while they are feeling physically and mentally overwhelmed (Kristanti and Effendy 2021; MacKenzie 2024). Situations where there is no clear right or wrong, combined with a lack of confidence in their decisions, further intensify this conflict (Hosseini et al. 2024; Rozo et al. 2025). For example, during the COVID‐19 pandemic, family caregivers felt torn between staying with their loved ones during hospitalisation or leaving them alone, according to institutional policies (Vachon et al. 2023). They face a conflict between staying and increasing the risk of spreading the virus to family and society, or leaving them alone in this challenging condition for the safety of others (Vachon et al. 2023). Another example of internal conflict is when family caregivers struggle with decisions regarding autonomy and safety, such as whether to intervene or not in situations like driving. They feel conflict between intervening and preventing their loved ones from driving to ensure safety, or not intervening and respecting their autonomy and independence (Lafrance et al. 2022).

#### 4.3.2 Self‐Directed Negative Emotions

Self‐directed negative emotions are feelings, such as guilt, shame, regret and self‐blame, that arise from individuals' self‐evaluations when they believe they have violated their own moral or ethical standards. These emotions are often classified as moral emotions, as they are closely tied to individuals' evaluations of their behaviour in relation to internalised moral standards (Tangney et al. 2007). Family caregivers frequently face negative self‐directed emotions due to ethically complex and challenging decision‐making processes (Hosseini et al. 2024; Rozo et al. 2025; Ullrich et al. 2020). They often experience feelings of failure and frustration when they are unable to provide the optimal care they want for their loved ones (Shewangizaw et al. 2023). Additionally, feelings of guilt, anger and low self‐esteem arise when caregivers find themselves in situations where they have no choice or control (Koenig 2004; Rozo et al. 2025). When they believe they have made the wrong decision, they experience fear, regret, self‐blame, confusion and self‐doubt (Rozo et al. 2025; Shewangizaw et al. 2023; Ullrich et al. 2020). The challenging situations and difficult caregiving decisions lead to emotions of anger, denial, shock, fear and endless grief (Hosseini et al. 2024). Moreover, the inability to balance multiple caregiving roles often results in feelings of inadequacy and frustration (Rozo et al. 2025).

#### 4.3.3 Powerlessness and Helplessness

Moral distress occurs when individuals have no power to act according to their moral and ethical beliefs (Jameton 1984). They experience situations where they face a lack of control and are forced to act in ways that conflict with their values (Ullrich et al. 2020). Additionally, they often feel powerless when they cannot help their loved ones get better or when they face a difficult decision‐making process (Gágyor et al. 2019; Paidipati et al. 2023). Helplessness emerges when family caregivers cannot provide the optimal care they want for their loved one, particularly in situations where they face financial restraints and a lack of resources (Rozo et al. 2025; Ullrich et al. 2020).

### 4.4 Antecedents

Antecedents are events that occur prior to the occurrence of the concept, and they are required for the concept to exist (Walker and Avant 2019). In this concept analysis, after reviewing the included articles, six antecedents occur before the occurrence of moral distress among family caregivers: caregiver burden, role conflict, complex decision‐making process, ethical dilemma and internal and external constraints.

#### 4.4.1 Caregiver Burden

To experience the challenges and burdens of caregiving, an individual must first take on the role of a family caregiver for someone with an acute or chronic condition. The sudden caregiving responsibilities expose them to physical, emotional and psychological burdens (Hosseini et al. 2024; Vachon et al. 2023). Caregivers must assist with activities of daily living, including bathing, feeding and mobility, which add to their physical strain (MacKenzie 2024). Moreover, they are responsible for coordinating health care appointments and managing medications and treatments, all of which contribute to stress and emotional burdens (MacKenzie 2024; Rozo et al. 2025).

#### 4.4.2 Role Conflict

Family caregivers face a lot of responsibilities, including their roles as parents, friends, spouses, family members and employees (Rozo et al. 2025; Ullrich et al. 2020; Vachon et al. 2023). These multiple roles make it difficult for them to manage and balance their time effectively (Gágyor et al. 2019; Koenig 2004; Rozo et al. 2025; Ullrich et al. 2020; Vachon et al. 2023). As a result, some family caregivers lose their jobs, while others become socially isolated due to these multiple roles and responsibilities (Shewangizaw et al. 2023). This ongoing role conflict creates significant pressure, particularly when caregivers feel they have not met expectations in their roles as caregivers (Vachon et al. 2023). MacKenzie (2024) emphasises that role conflict may also occur within the caregiving relationship itself, as caregivers often feel torn between performing physically and emotionally demanding caregiving tasks and preserving their identity as a loving spouse or family member. MacKenzie provides an example of this tension, describing a caregiver who refused to administer her partner's heparin injection because she could not bear to cause him pain. Although the task was medically necessary, the caregiver felt that performing it conflicted with her identity as a loving partner.

#### 4.4.3 Complex Decision‐Making Process

Family caregivers are often responsible for making critical care decisions for their loved ones, especially when they are entirely dependent and unable to decide for themselves (Ullrich et al. 2020; Weigel 2019). Common decisions include choosing between home care and nursing home placement, as well as making palliative care and resuscitation decisions (Hosseini et al. 2024; Koenig 2004; Kristanti and Effendy 2021). In addition, daily care‐related decisions, particularly those regarding when to seek help, further increase the complexity of decision‐making (Rozo et al. 2025). Uncertainty surrounding the illness trajectory leaves family caregivers unsure of how their loved one's condition will progress, making decisions even more complex and emotionally difficult (Rozo et al. 2025). The fear of making wrong decisions combined with a lack of confidence is overwhelming, especially since most caregivers do not have adequate knowledge and guidance (Hosseini et al. 2024; Kristanti and Effendy 2021; Ullrich et al. 2020).

#### 4.4.4 Ethical Dilemma

Family caregivers experience many morally undesirable situations when they feel that there is no choice that is entirely correct (Vachon et al. 2023). These situations involve conflicting ethical principles, such as autonomy versus protection, forcing caregivers to make choices and decisions that challenge their personal and moral values and beliefs (Hosseini et al. 2024; Koenig 2004; Vachon et al. 2023; Weigel 2019). End‐of‐life care is one of these situations where caregivers feel confused about the choices, whether to continue life‐prolonging treatment or transition to palliative care (Hosseini et al. 2024). Choosing between a peaceful death, human dignity, and the best interest of the person they care for is challenging (Hosseini et al. 2024).

#### 4.4.5 Internal Constraints

These include the challenges that arise within the caregivers and make them feel inadequate, overwhelmed and conflicted (Ullrich et al. 2020). One of these challenges is insufficient knowledge and inadequate information (Ullrich et al. 2020). Most family caregivers have suddenly found themselves responsible for complex tasks and responsibilities without sufficient training and support (Ullrich et al. 2020). Furthermore, time constraints further exacerbate the distress (MacKenzie 2024; Shewangizaw et al. 2023; Ullrich et al. 2020).

#### 4.4.6 External Constraints

These include financial barriers, such as increased medical costs and limited insurance coverage, which create significant stress (Koenig 2004; Rozo et al. 2025; Shewangizaw et al. 2023). As a result of these barriers, caregivers experience negative self‐directed feelings when they cannot afford the cost of providing their loved one with the perfect care and treatment they desire (Shewangizaw et al. 2023). Moreover, institutional rules and policies also serve as barriers for caregivers to do what they believe is morally right (Vachon et al. 2023). During the COVID‐19 pandemic, many caregivers were prohibited from visiting their loved ones due to institutional policies, and these situations intensified their emotional burdens (Vachon et al. 2023). In addition, limited access to healthcare services, inadequate resources, a shortage of formal care facilities, and the high cost of nursing homes, palliative care, and hospice leave caregivers with few options (Koenig 2004; Rozo et al. 2025).

### 4.5 Consequences

Consequences are the events that happen as a result or outcome of the concept (Walker and Avant 2019). In this analysis, four main consequences were identified after reviewing the included articles: long‐term health effects, social withdrawal and isolation, burnout and moral residue.

#### 4.5.1 Long‐Term Health Effects (Physical and Psychological)

Family caregivers experiencing prolonged or unresolved moral distress may develop significant long‐term physical and psychological health effects (Rozo et al. 2025). The physical impact includes chronic fatigue, exhaustion and sleep disturbances due to prolonged stress (Voultsos et al. 2024). Many caregivers, as a result of the ongoing caregiving burden and unresolved ethical dilemmas, experience heightened levels of anxiety and persistent sadness (Rozo et al. 2025; Voultsos et al. 2024).

#### 4.5.2 Social Withdrawal and Isolation

Family caregivers manage multiple responsibilities and roles, and morally distressing situations. Many struggle to balance these roles with their social lives (Koenig 2004; Ullrich et al. 2020). Due to increased caregiving demands, many caregivers find it challenging to maintain their social relationships as they did in the past (Rozo et al. 2025). Those caring for ageing parents often prioritise caregiving responsibilities over their social and familial connections. This leads to significant disruptions in personal relationships, including marital conflict and divorce (Rozo et al. 2025).

#### 4.5.3 Burnout

Family caregivers experience two aspects of burnout as a result of moral distress. The first occurs when the caregiver becomes emotionally and physically exhausted, drained, and unable to tolerate the demands of caregiving. In this case, they may feel unable to continue their caregiving role and seek alternative care options such as home nurses, institutional care or hospice (Weigel 2019). The second aspect of burnout occurs when caregivers need to adjust their work due to a conflict between caregiving responsibilities and work obligations (Rozo et al. 2025). These adjustments include changing shifts, reducing work hours, asking for unpaid leave or even leaving their jobs (Rozo et al. 2025). Many family caregivers struggle to balance the complex demands of caregiving, family and work, leading them to leave their jobs (Gágyor et al. 2019; Koenig 2004; Ullrich et al. 2020; Vachon et al. 2023).

#### 4.5.4 Moral Residue

Some family caregivers repeatedly face morally challenging situations and difficult decisions (Ullrich et al. 2020). When they make a decision that conflicts with their moral values and beliefs over an extended period of time, they may experience unresolved and prolonged distress, known as moral residue (Ullrich et al. 2020). This enduring psychological distress results from the prolonged persistence of self‐directed negative emotions such as guilt, regret or shame that remain long after a morally distressing situation has passed (Shewangizaw et al. 2023; Ullrich et al. 2020).

### 4.6 Empirical Referents

The empirical referents are directly related to the means of recognising and measuring the attributes and characteristics of moral distress among family caregivers. Determining the existence of moral distress in the real world involves identifying several observable characteristics. These referents or characteristics manifest through verbal expressions of distress, behavioural changes and physiological responses. Common indicators include negative self‐directed emotions such as feelings of guilt, anger, low self‐esteem, frustration, fear, regret, self‐blame, anxiety, sadness, confusion, self‐doubts and sleep problems (Rozo et al. 2025; Shewangizaw et al. 2023; Ullrich et al. 2020). Additionally, powerlessness and helplessness can arise due to ethical dilemmas, complex decision‐making processes and internal conflicts (Gágyor et al. 2019; MacKenzie 2024; Paidipati et al. 2023). Caregivers may also exhibit social withdrawal due to the difficulty of balancing their multiple roles and caregiving responsibilities and even the loss of their jobs (Koenig 2004; Ullrich et al. 2020; Weigel 2019).

### 4.7 Identify Model Case

The model case is an example from real‐life, literature or construction by the researcher (Walker and Avant 2019). It demonstrates all the defining attributes and characteristics of the concept of moral distress among family caregivers and works as a model. The author constructed the following model case.

### 4.8 Model Case

Ali is a 52‐year‐old male taking care of his wife, Laila, who experienced a stroke 1 year ago. Despite months of rehabilitation, her condition has not improved. Recently, Laila was admitted to the hospital because of recurrent infections, bedsores and difficulty breathing. Ali faced a difficult medical decision of whether to go with aggressive medical treatment and intervention, such as mechanical ventilation, or to go to palliative care since her condition did not improve. The doctor explained to Ali that his wife's condition is difficult and that the intensive treatment and intervention may prolong her life but not improve her quality of life and may increase her suffering. Ali feels a deep internal conflict between his ethical obligations, values and beliefs that his wife deserves every possible chance to live versus the reality that continued medical treatment may only add to her suffering and increase harm more than good. He experiences intense negative feelings, including frustration, sadness, anger, self‐doubt, shame, anxiety, helplessness and powerlessness as he struggles to choose what is best for his wife. His exhaustion and inability to balance caregiving responsibilities, family obligations, and his job make the decision even harder. With time, he decided to go with palliative care. However, after he made this decision, he struggled with prolonged negative feelings of guilt, regret and deep sadness. He constantly thinks that he made the wrong decision, blames himself for her death, and feels like he failed to be a good husband.

### 4.9 Contrary Case

The cases that do not reflect the defining attributes are called contrary cases (Walker and Avant 2019). The author constructed the following contrary case. Maria is a 55‐year‐old woman taking care of her husband, Jad, who was diagnosed with terminal cancer. Jad expresses his clear desire to spend the last days of his life with his family at home without hospitalisation or chemotherapy. Maria discussed the condition of her husband with the physicians, and they told her that the cancer had already spread extensively. The physician told Maria that chemotherapy was an option, but it would not guarantee an improvement in his health. Maria discussed the situation and Jad's desire with her family members, and they all made the decision to respect their father's wishes. Maria feels at peace with this decision because she knows that this is the desire of her husband and that all family members are in agreement with this decision. She experiences normal feelings of sadness and anxiety regarding her husband's condition with no feelings of regret or guilt about her decision. She provides emotional and physical support for her husband in his final days. This case lacks the key defining attributes of moral distress. Instead, she has full support from her family and medical team, allowing her to provide care without distress or regret.

### 4.10 Borderline Case

The cases that contain most of the defining attributes but not all of them are called borderline cases (Walker and Avant 2019). Tom is a 33‐year‐old man taking care of his mother, who has been diagnosed with Alzheimer's disease. She is completely dependent on him for daily care, and in recent months, her condition has worsened. She became more agitated, aggressive, and confused, and refused to eat. As a result, she lost weight, and Tom took her to the doctor, who diagnosed her with malnutrition and dehydration. The doctor suggested putting in a feeding tube because they had already tried all the other available options, but she still refused to eat. However, Tom's mother repeatedly expresses her wish not to receive medical interventions, saying, ‘I just want to die in peace’. Tom became conflicted between respecting his mother's wishes and his fear that her health would deteriorate further without this intervention. He felt stressed, sad, and doubtful, struggling with whether to go with her wishes or proceed with the feeding tube. After he had discussed the situation with her doctor, he decided to proceed with the feeding tube. While he experienced feelings of guilt, he reassured himself that he had made the right decision for her health. When his mother woke up and realised she had a feeding tube, she began crying and screaming, which sometimes made him feel guilty and regretful. However, after a short time, he felt hopeful that he had made the right decision as her condition stabilised.

## 5 The Final Definition

Based on the findings of this analysis, a conceptual definition of moral distress in the context of family caregiving is described as follows:

The emotional and psychological turmoil that arises when family caregivers experience ethically challenging and complex decision‐making situations that prevent them from acting according to their moral values and beliefs is characterised by self‐directed negative feelings and emotions, internal conflicts, and feelings of powerlessness and helplessness. It can lead to long‐term consequences such as long‐term health effects (physical and psychological), social isolation, burnout and moral residue.

## 6 Discussion

The findings from this concept analysis contribute to an understanding of moral distress among family caregivers. Although several concept analyses have explored moral distress, none have specifically examined this concept within the context of family caregiving. Some concept analysis papers connect the concept of moral distress to healthcare providers, such as critical care nurses (Cooke et al. 2022), nursing students (Rebecca 2025), midwifery (Foster et al. 2022) and moral distress in pain management (Byma 2025). The primary purpose of this analysis was to examine and define the concept of moral distress in the context of family caregiving.

This concept analysis identified both similarities and differences in how family caregivers experience moral distress compared to other populations in terms of defining attributes, antecedents and consequences. After reviewing the included articles, the defining attributes and characteristics used in the literature to identify moral distress among family caregivers are self‐directed negative emotions, internal conflict and feelings of powerlessness and helplessness. These attributes align with those reported by Cooke et al. (2022) and Foster et al. (2022), who found that nurses and midwives experiencing moral distress exhibited negative feelings and emotions, as well as a lack of power. However, a distinctive attribute identified by Byma's (2025) work on moral distress in pain management was the feeling of ownership over the patient's pain. Nurses in this context knew they had a clear intervention (e.g., analgesia) to manage patients' pain but were constrained from administering it, leading to moral distress. In contrast, family caregivers often lack a clear or accessible solution to alleviate their loved one's suffering, which distinguishes their experience of moral distress from that of nurses in pain management contexts.

Six antecedents were identified in this analysis as sources of moral distress among family caregivers: caregiving burden, role conflict, ethical dilemma, complex decision‐making process, internal constraints and external constraints. One of these antecedents that is similar across all contexts is the external constraints, including a lack of resources and institutional barriers (Byma 2025; Cooke et al. 2022; Foster et al. 2022). While some antecedents are shared, others differ. For example, unlike family caregivers, a key antecedent for moral distress among critical care nurses is perceptions of futile care (Cooke et al. 2022). In contrast, family caregivers often struggle with the complex decision‐making process (Hosseini et al. 2024; Kristanti and Effendy 2021).

This analysis also highlighted the negative outcomes and consequences that family caregivers experience as a result of experiencing moral distress, including social isolation, burnout, long‐term health effects (physical and psychological), as well as moral residue. While burnout and long‐term health effects (physical and psychological) are common to both healthcare providers and family caregivers (Byma 2025; Cooke et al. 2022; Foster et al. 2022). Social isolation is a distinct consequence that is primarily associated with family caregivers. One possible reason for this distinct consequence is that family caregiving is like a full‐time job (AARP and National Alliance for Caregiving 2020). Unlike nurses, who typically work defined shifts, family caregivers often face continuous demands, which increases their risk of social withdrawal and isolation.

### 6.1 Limitations

This concept analysis has limitations regarding the included studies and regarding the analysis process itself. First, nine of the included articles were qualitative studies, and the remaining three were theoretical and perspective papers, which limit the generalizability of the results. Perhaps the reason for the exclusive focus on qualitative papers is that no instrument is available to measure moral distress among family caregivers quantitatively. Second, only five articles explicitly discussed moral distress among family caregivers. The other seven articles primarily discussed ethical decision‐making challenges and ethical dilemmas. While these concepts overlap with moral distress, they may not fully capture its defining attributes and characteristics. Third, the articles that explicitly discuss moral distress involve family caregivers of individuals with cancer, dementia and Alzheimer's. Notably, none of these articles refer to family caregivers of stroke survivors who face significant caregiving burdens.

### 6.2 Implications for Research and Practice

The findings of this concept analysis highlight some very important implications for future research and practice. Future research should investigate moral distress among family caregivers in greater detail. Most specifically, there is a gap in studying ethical challenges and moral distress among caregivers of stroke survivors. In addition, instruments specifically designed to measure moral distress in family caregivers need to be developed and validated. Once these instruments are developed, quantitative research is recommended to assess the prevalence and severity of moral distress among family caregivers.

In practice, healthcare providers, including physicians and nurses, should be aware of the ethical dilemmas that family caregivers experience, as well as their effects on the caregivers' lives and health. Support systems such as training and educational programmes should be designed to help family caregivers build the skills and knowledge needed to manage ethically challenging situations. Healthcare providers also play an important role in linking caregivers to helpful resources, including counselling, peer support groups and community‐based services. Such initiatives will not only improve the well‐being of caregivers but also enhance the quality of patient care and promote more ethical, collaborative healthcare environments.

## 7 Conclusion

Moral distress among family caregivers is a significant and underrecognized issue. It affects their well‐being and the quality of care they provide. This paper offered a structured examination and a clear conceptual definition of moral distress within the context of family caregiving. The conceptual definition will help operationalise moral distress in the context of family caregivers by developing relevant instruments. Researchers can use these instruments to measure, study and develop interventions that support family caregivers.

## Funding

The authors have nothing to report.

## Ethics Statement

The authors have nothing to report.

## Consent

The authors have nothing to report.

## Conflicts of Interest

The authors declare no conflicts of interest.

## Data Availability

No new data were generated or analysed in support of this research. Data sharing is not applicable to this article.

## References

[jan70444-bib-0001] AARP , and National Alliance for Caregiving . 2020. Caregiving in the United States 2020: Full Report. AARP Public Policy Institute. https://www.aarp.org/ppi/info‐2020/caregiving‐in‐the‐united‐states.html.

[jan70444-bib-0002] Alzheimer's Association . 2024. “2024 Alzheimer's Disease Facts and Figures.” Alzheimer's & Dementia 20, no. 5: 3708–3821. 10.1002/alz.13809. PMC11095490 38689398

[jan70444-bib-0003] American Psychological Association . 2011. “Caregiving Facts. APA Psychology and Aging.” https://www.apa.org/pi/about/publications/caregivers/faq.

[jan70444-bib-0005] American Stroke Association . 2023. “Let's Talk About Being a Caregiver for a Stroke Survivor.” https://www.stroke.org.

[jan70444-bib-0006] Barlem, E. L. D. , and F. R. S. Ramos . 2015. “Constructing a Theoretical Model of Moral Distress.” Nursing Ethics 22, no. 5: 608–615. 10.1177/0969733014551595. 25366998

[jan70444-bib-0007] Bilić, J. , L. Skokandić , and L. Puljak . 2022. “Anticipatory Grief and Experience of Providing At‐Home Palliative Care Among Informal Caregivers of Spouses in Croatia: A Qualitative Study.” BMC Palliative Care 21: 199. 10.1186/s12904-022-01093-1. 36397096 PMC9672539

[jan70444-bib-0008] Byma, E. A. 2025. “Concept Analysis of Moral Distress in Pain Management.” Pain Management Nursing 26, no. 2: 206–211. 10.1016/j.pmn.2024.10.014. 39547855

[jan70444-bib-0009] Campbell, S. M. , C. M. Ulrich , and C. Grady . 2016. “A Broader Understanding of Moral Distress.” American Journal of Bioethics 16, no. 12: 2–9. 10.1080/15265161.2016.1239782. 27901442

[jan70444-bib-0010] Centers for Disease Control and Prevention . 2018. Caregiving for Family and Friends—A Public Health Issue. U.S. Department of Health and Human Services. https://www.cdc.gov/healthy‐aging‐data/media/pdfs/caregiver‐brief‐508.pdf.

[jan70444-bib-0011] Choe, K. , Y. Kang , and Y. Park . 2015. “Moral Distress in Critical Care Nurses: A Phenomenological Study.” Journal of Advanced Nursing 71, no. 7: 1684–1693. 10.1111/jan.12638. 25688835

[jan70444-bib-0012] Cooke, S. , R. Booth , and K. Jackson . 2022. “Moral Distress in Critical Care Nursing Practice: A Concept Analysis.” Nursing Forum 57, no. 6: 1478–1483. 10.1111/nuf.12786. 35962765

[jan70444-bib-0013] Corley, M. C. , R. K. Elswick , M. Gorman , and T. Clor . 2001. “Development and Evaluation of a Moral Distress Scale.” Journal of Advanced Nursing 33, no. 2: 250–256. 10.1111/j.1365-2648.2001.01658.x. 11168709

[jan70444-bib-0014] Deschenes, S. , M. Gagnon , T. Park , and D. Kunyk . 2020. “Moral Distress: A Concept Clarification.” Nursing Ethics 27, no. 4: 1127–1146. 10.1177/0969733020909523. 32249662

[jan70444-bib-0015] Dudzinski, D. M. 2016. “Navigating Moral Distress Using the Moral Distress Map.” Journal of Medical Ethics 42, no. 5: 321–324. 10.1136/medethics-2015-103156. 26969723

[jan70444-bib-0016] Epstein, E. G. , and A. B. Hamric . 2009. “Moral Distress, Moral Residue, and the Crescendo Effect.” Journal of Clinical Ethics 20, no. 4: 330–342. 20120853

[jan70444-bib-0017] Family Caregiver Alliance . n.d. “Definitions.” https://www.caregiver.org/resource/definitions‐0/.

[jan70444-bib-0018] Foster, W. , L. McKellar , J. Fleet , and L. Sweet . 2022. “Moral Distress in Midwifery Practice: A Concept Analysis.” Nursing Ethics 29, no. 2: 364–383. 10.1177/09697330211023983. 34538155

[jan70444-bib-0019] Gágyor, I. , A. Heßling , S. Heim , A. Frewer , F. Nauck , and W. Himmel . 2019. “Ethical Challenges in Primary Care: A Focus Group Study With General Practitioners, Nurses and Informal Caregivers.” Family Practice 36, no. 2: 225–230. 10.1093/fampra/cmy060. 29931146 PMC6425460

[jan70444-bib-0020] Gurley Nettles, T. C. 2024. “Examining Factors That Contribute to Poststroke Depression Within the Family Caregiver and Care Recipient Dyadic Experience.” Perspectives of the ASHA Special Interest Groups 9, no. 6: 1853–1867. 10.1044/2024_PERSP-23-00294.

[jan70444-bib-0021] Hardingham, L. B. 2004. “Integrity and Moral Residue: Nurses as Participants in a Moral Community.” Nursing Philosophy 5, no. 2: 127–134. 10.1111/j.1466-769X.2004.00160.x. 15189553

[jan70444-bib-0022] Hosseini, S. E. , A. N. Narabadi , A. Abbasi , S. Joolaee , N. Sheikhzakaryaee , and M. Shali . 2024. “Exploring the Ethical Decision‐Making Experience of Caregivers of End Stage Cancer Patients in Iran: A Phenomenological Study.” BMC Medical Ethics 25, no. 1: 130. 10.1186/s12910-024-01131-y. 39543594 PMC11566908

[jan70444-bib-0023] International Alliance of Carer Organisations . 2018. Carer Facts. International Alliance of Carer Organisations. https://carersworldwide.org/blog/global‐carers‐statistics.

[jan70444-bib-0024] James, C. O. 2021. Moral Distress in the Care of People Living With Moderate to Advanced Dementia: A Narrative Exploration of Family Carers' Experience of Home‐Based Care Provision Towards the End of Life. Doctoral Dissertation. Lancaster University. ProQuest Dissertations & Theses Global. https://www.proquest.com/docview/2665129447/abstract/C5220D4A02C74787PQ/1.

[jan70444-bib-0025] Jameton, A. 1984. “Nursing Practice.” In The Ethical Issues, 51336. Eweb. https://repository.library.georgetown.edu/handle/10822/800986.

[jan70444-bib-0026] Jaracz, K. , B. Grabowska‐Fudala , J. Jaracz , et al. 2024. “Caregiver Burden After Stroke: A 10‐Year Follow‐Up Study of Polish Caregivers for Stroke Patients.” BMC Nursing 23, no. 1: 589. 10.1186/s12912-024-02251-x. 39183261 PMC11346017

[jan70444-bib-0027] Jaskela, S. , J. Guichon , S. Page , and I. Mitchell . 2018. “Social Workers' Experience of Moral Distress.” Canadian Social Work Review 35, no. 1: 91–107. 10.7202/1051104ar.

[jan70444-bib-0028] Koenig, T. L. 2004. “From the Woman's Viewpoint: Ethical Dilemmas Confronted by Women as Informal Caregivers of Frail Elders.” Families in Society 85, no. 2: 236–242. 10.1606/1044-3894.319.

[jan70444-bib-0029] Kristanti, M. S. , and C. Effendy . 2021. “Common Ethical Dilemmas of Family Caregivers of Palliative Patients in Indonesia.” Belitung Nursing Journal 7, no. 3: 246–250. 10.33546/bnj.1457. 37469338 PMC10353594

[jan70444-bib-0063] Kristanti, M. S. , C. Effendy , A. Utarini , M. Vernooij‐Dassen , and Y. Engels . 2019. “The Experience of Family Caregivers of Patients with Cancer in an Asian Country: A Grounded Theory Approach.” Palliative Medicine 33, no. 6: 676–684. 10.1177/0269216319833260. 30916614 PMC6537031

[jan70444-bib-0064] Kristanti, M. S. , Y. Engels , C. Effendy , A. Utarini , and M. Vernooij‐Dassen . 2018. “Comparison of the Lived Experiences of Family Caregivers of Patients with Dementia and of Patients with Cancer in Indonesia.” International Psychogeriatrics 30, no. 6: 903–914. 10.1017/S1041610217001508. 28870266 PMC6088529

[jan70444-bib-0030] Lafrance, M. N. , E. Dreise , L. Gouliquer , and C. Poulin . 2022. ““We're Not Doing It to Be Nasty”: Caregivers' Ethical Dilemmas in Negotiating Driving Safety With Older Adults.” Canadian Journal on Aging/La Revue Canadienne du Vieillissement 41, no. 1: 7–14. 10.1017/S0714980820000409. 33397532

[jan70444-bib-0031] Lawson, S. 2022. “Caregiver Decision‐Making Process Analysis: A Grounded Theory Approach.” American Journal of Occupational Therapy 76: 7610505051p1. 10.5014/ajot.2022.76S1-PO51.

[jan70444-bib-0032] Lewandowska, A. 2022. “Parents and Their Children in the Face of Cancer: Parents' Expectations, Changes in Family Functioning in the Opinion of Caregivers of Children With Neoplastic Diseases—Further Studies.” Children 9, no. 10: 1562. 10.3390/children9101562. 36291498 PMC9600004

[jan70444-bib-0033] Lindeza, P. , M. Rodrigues , J. Costa , M. Guerreiro , and M. M. Rosa . 2024. “Impact of Dementia on Informal Care: A Systematic Review of Family Caregivers' Perceptions.” BMJ Supportive & Palliative Care 14, no. e1: e38–e49. 10.1136/bmjspcare-2020-002242. 33055092

[jan70444-bib-0034] Lobo, E. H. , M. Abdelrazek , J. Grundy , et al. 2021. “Caregiver Engagement in Stroke Care: Opportunities and Challenges in Australia and Denmark.” Frontiers in Public Health 9: 758808. 10.3389/fpubh.2021.758808. 34900907 PMC8661098

[jan70444-bib-0035] MacKenzie, J. 2024. “Caregiving and Role Conflict Distress.” Clinical Ethics 19, no. 2: 136–142. 10.1177/14777509231218565.

[jan70444-bib-0066] Martin, S. S. , A. W. Aday , Z. I. Almarzooq , et al. 2024. “Heart Disease and Stroke Statistics—2024 Update: A Report of U.S. and Global Data from the American Heart Association.” Circulation 149, no. 8. 10.1161/CIR.0000000000001209. PMC12146881 38264914

[jan70444-bib-0036] McCurley, J. L. , C. J. Funes , E. L. Zale , et al. 2019. “Preventing Chronic Emotional Distress in Stroke Survivors and Their Informal Caregivers.” Neurocritical Care 30, no. 3: 581–589. 10.1007/s12028-018-0641-6. 30421266 PMC6958510

[jan70444-bib-0037] Miljeteig, I. , R. Førde , K. I. Rø , F. Bååthe , and B. Bringedal . 2023. “Resource Conflicts Leading to Moral Distress: A Longitudinal Study Among Physicians in Norway.” *medRxiv*, 2023‐09. 10.1101/2023.09.29.23295833.

[jan70444-bib-0038] Moher, D. , A. Liberati , J. Tetzlaff , D. G. Altman , and The PRISMA Group . 2009. “Preferred Reporting Items for Systematic Reviews and Meta‐Analyses: The PRISMA Statement.” PLoS Medicine 6, no. 7: e1000097. 10.1371/journal.pmed.1000097. 19621072 PMC2707599

[jan70444-bib-0040] National Cancer Institute . 2024. Family Caregivers in Cancer: Support and Challenges (PDQ Patient Version). National Cancer Institute, U.S. Department of Health and Human Services. https://www.cancer.gov/about‐cancer/coping/family‐friends/family‐caregivers‐pdq.

[jan70444-bib-0041] Ong, R. S. R. , R. S. M. Wong , R. C. H. Chee , et al. 2022. “A Systematic Scoping Review Moral Distress Amongst Medical Students.” BMC Medical Education 22, no. 1: 466. 10.1186/s12909-022-03515-3. 35710490 PMC9203147

[jan70444-bib-0042] Page, M. J. , J. E. McKenzie , P. M. Bossuyt , et al. 2021. “The PRISMA 2020 Statement: An Updated Guideline for Reporting Systematic Reviews.” BMJ 372: n71. 10.1136/bmj.n71. 33782057 PMC8005924

[jan70444-bib-0043] Paidipati, C. P. , A. M. Foxwell , K. Mooney‐Doyle , D. Tiller , J. Pinto‐Martin , and C. M. Ulrich . 2023. “Caregiver Perspectives on the Benefits, Burdens, and Moral Distress of Participation in Cancer Clinical Trials.” Journal of Family Nursing 29, no. 1: 89–98. 10.1177/10748407221098187. 35611586

[jan70444-bib-0044] Rebecca, T. 2025. “Student Nurses Experiences of Moral Distress: A Concept Analysis.” Journal of Advanced Nursing 81, no. 2: 730–748. 10.1111/jan.16370. 39101378 PMC11729407

[jan70444-bib-0045] Rozo, A. J. , M. Schneiders , M. R. Palombit , et al. 2025. “Moral Distress Among Family Caregivers of People With Cancer in Palliative Care: A Qualitative Study.” Canadian Oncology Nursing Journal/Revue Canadienne de Soins Infirmiers en Oncologie 35, no. 2: 361–367. 10.5737/23688076352361 PMC12379905 40873897

[jan70444-bib-0046] Sajwani‐Merchant, Z. , D. Behan , C. Swank , and K. Daniel . 2023. “Caregiver Experiences of Social Support Following Stroke.” Journal of Stroke and Cerebrovascular Diseases 32, no. 9: 107253. 10.1016/j.jstrokecerebrovasdis.2023.107253. 37544057

[jan70444-bib-0067] Schulz, R. , and J. Eden , eds. 2016. “Family Caregiving Roles and Impacts.” In Families Caring for an Aging America. National Academies Press. https://www.nationalacademies.org/publications/23606. 27905704

[jan70444-bib-0047] Sheehan, O. C. , W. E. Haley , V. J. Howard , J. Huang , J. D. Rhodes , and D. L. Roth . 2021. “Stress, Burden, and Well‐Being in Dementia and Nondementia Caregivers: Insights From the Caregiving Transitions Study.” Gerontologist 61, no. 5: 670–679. 10.1093/geront/gnaa108. 32816014 PMC8276607

[jan70444-bib-0048] Shewangizaw, S. , W. Fekadu , C. Sackley , and A. Alem . 2023. “Life After Stroke: Exploring Social and Psychological Consequences of Stroke Survivors and Their Caregivers.” Ethiopian Medical Journal 61, no. 4: 4. 10.4314/emj.v61i4.3.

[jan70444-bib-0049] Stork, R. , M. Martone , P. Osterman , T. A. Savage , and D. Mukherjee . 2018. “The Family Caregiving Dilemma.” PM&R 10, no. 1: 90–96. 10.1016/j.pmrj.2017.12.001. 29413122

[jan70444-bib-0050] Sugawara, N. , N. Yasui‐Furukori , K. Maruo , K. Shimoda , and T. Sumiyoshi . 2023. “Predictors of Psychological Distress and Sleep Deprivation in Caregivers of Stroke Survivors.” Journal of Stroke and Cerebrovascular Diseases: The Official Journal of National Stroke Association 32, no. 1: 106899. 10.1016/j.jstrokecerebrovasdis.2022.106899. 36403364

[jan70444-bib-0051] Tangney, J. P. , J. Stuewig , and D. J. Mashek . 2007. “Moral Emotions and Moral Behavior.” Annual Review of Psychology 58: 345–372. 10.1146/annurev.psych.56.091103.070145. PMC3083636 16953797

[jan70444-bib-0052] Thumvichit, A. 2023. “‘I'm Aware of That, but …’: Breaking the Silence on Moral Distress Among Language Teachers.” Language, Culture and Curriculum 36, no. 3: 343–360. 10.1080/07908318.2023.2189268.

[jan70444-bib-0053] Ullrich, A. , M. Theochari , C. Bergelt , et al. 2020. “Ethical Challenges in Family Caregivers of Patients With Advanced Cancer—A Qualitative Study.” BMC Palliative Care 19, no. 1: 70. 10.1186/s12904-020-00573-6. 32423444 PMC7236546

[jan70444-bib-0054] Vachon, M. , A. Guité‐Verret , D. Ummel , and D. Girard . 2023. “‘I Couldn't’: A Phenomenological Exploration of Ethical Tensions Experienced by Bereaved Family Members During the Pandemic.” International Journal of Qualitative Studies on Health and Well‐Being 18, no. 1: 2186337. 10.1080/17482631.2023.2186337. 36919516 PMC10026767

[jan70444-bib-0055] Váchová, M. 2019. “Development of a Tool for Determining Moral Distress Among Teachers in Basic Schools.” Pedagogika 69, no. 4: 4. 10.14712/23362189.2019.1524.

[jan70444-bib-0065] Voultsos, P. , M. Arabatzi , M. Deligianni , and A. K. Tsaroucha . 2024. “Extending the Concept of Moral Distress to Parents of Infants Hospitalized in the NICU: A Qualitative Study in Greece.” BMC Psychology 12, no. 1: 291. 10.1186/s40359-024-01793-8. 38790072 PMC11127332

[jan70444-bib-0056] Vu, M. , R. Mangal , T. Stead , C. Lopez‐Ortiz , and L. Ganti . 2022. “Impact of Alzheimer's Disease on Caregivers in the United States.” Health Psychology Research 10, no. 3: 37454. 35999976 10.52965/001c.37454 PMC9392839

[jan70444-bib-0057] Walker, L. O. , and K. C. Avant . 2019. Strategies for Theory Construction in Nursing. 6th ed. Pearson.

[jan70444-bib-0058] Weigel, C. 2019. “Caregiving and Moral Distress for Family Caregivers During Early‐Stage Alzheimer's Disease.” International Journal of Feminist Approaches to Bioethics 12, no. 2: 74–91. 10.3138/ijfab.12.2.05.

[jan70444-bib-0059] Wilkinson, J. M. 1987. “Moral Distress in Nursing Practice: Experience and Effect.” Nursing Forum 23, no. 1: 16–29. 10.1111/j.1744-6198.1987.tb00794.x. 3454003

[jan70444-bib-0060] Wilson, J. 1963. Thinking With Concepts. Cambridge University Press.

[jan70444-bib-0061] World Health Organisation . 2025. WHO and Noora Health Begin Collaboration to Strengthen Support for Family Caregivers. World Health Organisation. https://www.who.int/news/item/29‐07‐2025‐who‐and‐noora‐health‐begin‐collaboration‐to‐strengthen‐support‐for‐family‐caregivers.

[jan70444-bib-0062] Yildiz, E. 2019. “Ethics in Nursing: A Systematic Review of the Framework of Evidence Perspective.” Nursing Ethics 26, no. 4: 1128–1148. 10.1177/0969733017734412. 29166840

